# Pulmonary Thromboembolism Secondary to Ovarian Vein Thrombosis in a Patient With Left-Sided Inferior Vena Cava Following Laparoscopic Gallbladder Bed Resection

**DOI:** 10.7759/cureus.107832

**Published:** 2026-04-27

**Authors:** Hideaki Sato, Shuichi Aoki, Naoki Rikiyama, Masamichi Mizuma, Michiaki Unno

**Affiliations:** 1 Surgery, Tohoku University, Sendai, JPN

**Keywords:** gallbladder tumor, laparoscopic gallbladder bed resection, left-sided inferior vena cava, ovarian vein thrombosis, pulmonary thromboembolism

## Abstract

Left-sided inferior vena cava (IVC) is a rare vascular anomaly that can alter venous hemodynamics and potentially increase the risk of deep vein thrombosis and pulmonary thromboembolism (PTE). Ovarian vein thrombosis (OVT) is typically associated with obstetric or gynecologic conditions and is rarely reported as a complication of gastrointestinal surgery. Here, we report a unique case of PTE resulting from OVT in a patient with left-sided IVC who underwent laparoscopic gallbladder bed resection.

A 79-year-old woman with a preoperative diagnosis of left-sided IVC was under surveillance for gallbladder adenomyomatosis. Due to progressive gallbladder wall thickening and suspected carcinoma, she underwent laparoscopic gallbladder bed resection. The procedure included station #16b1 lymph node sampling, during which the right ovarian vein could not be identified. The operation was performed in the reverse Trendelenburg position and lasted 268 min. On postoperative day five, routine contrast-enhanced computed tomography (CT) revealed a right OVT and subsequent PTE. The patient was promptly treated with anticoagulation therapy, initially with heparin followed by rivaroxaban. She was discharged on postoperative day 13 without further complications. A pre-discharge CT scan showed improvement in the PTE, although the OVT remained largely unchanged. Final pathological examination revealed a benign hyperplastic polyp and adenomyomatosis.

This rare case suggests that left-sided IVC may predispose patients to postoperative thrombotic complications, such as OVT, leading to PTE even after minimally invasive abdominal surgery. This underscores the importance of meticulous preoperative vascular assessments to identify anatomical variations. Furthermore, heightened clinical vigilance for venous thromboembolism, including OVT, is warranted in such patients. Postoperative thromboprophylaxis may be prudent in this population. This case highlights a novel association between left-sided IVC, OVT, and PTE in the context of gastrointestinal surgery.

## Introduction

The left-sided inferior vena cava (IVC) is a rare congenital anomaly where the IVC is positioned on the left side of the aorta. It typically crosses to the right at the level of the left renal vein to join the normal right-sided IVC [1,2]. Compared with the typical anatomy, left-sided IVC is associated with altered hemodynamics, potentially increasing the risk of deep vein thrombosis (DVT) and pulmonary thromboembolism (PTE) [1,3,4].

Ovarian vein thrombosis (OVT), which is characterized by thrombus formation within the ovarian vein, is the most common postpartum complication, particularly after cesarean section, and may be associated with pelvic infections, malignancies, and coagulation disorders [5,6]. A recent prospective cohort study has clarified that symptomatic postpartum OVT typically presents with a distinct clinical profile, where abdominal pain and fever are almost universally reported as the primary clinical manifestations, often occurring within the first two weeks after delivery [7]. The pathophysiology is fundamentally rooted in Virchow’s triad, where mechanical manipulation during surgery and postoperative systemic inflammation significantly elevate the risk of thrombosis [8]. While OVT is not widely recognized as a complication of gastrointestinal surgery, it is significant because it can lead to PTE, a potentially life-threatening condition [5,9].

Here, we present a rare case of PTE secondary to OVT in a patient with left-sided IVC who underwent laparoscopic gallbladder bed resection. This case highlights the importance of recognizing left-sided IVC as a potential risk factor for thromboembolic complications in minimally invasive abdominal surgery.

## Case presentation

**Figure 1:**
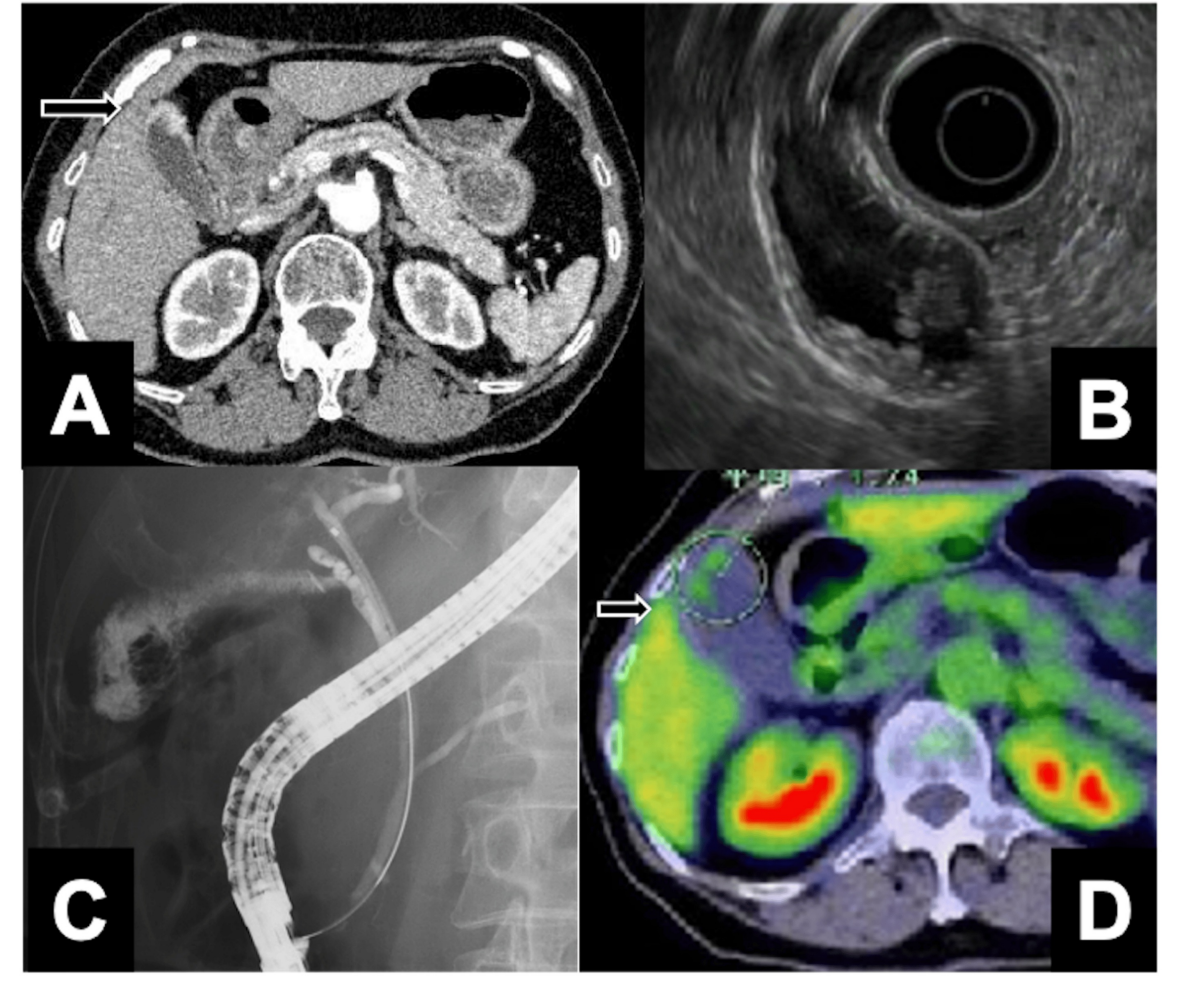
Preoperative imaging findings A: Contrast-enhanced CT revealed wall thickening at the fundus of the gallbladder (arrow), raising suspicion of gallbladder carcinoma. B: Endoscopic ultrasonography (EUS) suggested gallbladder carcinoma. C: Endoscopic retrograde cholangiopancreatography (ERCP) demonstrated a pancreaticobiliary maljunction without significant dilatation of the common bile duct. The amylase level of the bile juice collected during the ERCP was 200,111 IU/L. D: Positron emission tomography with fluorodeoxyglucose (FDG-PET) demonstrated mild FDG uptake in the tumor (arrow).

**Figure 2:**
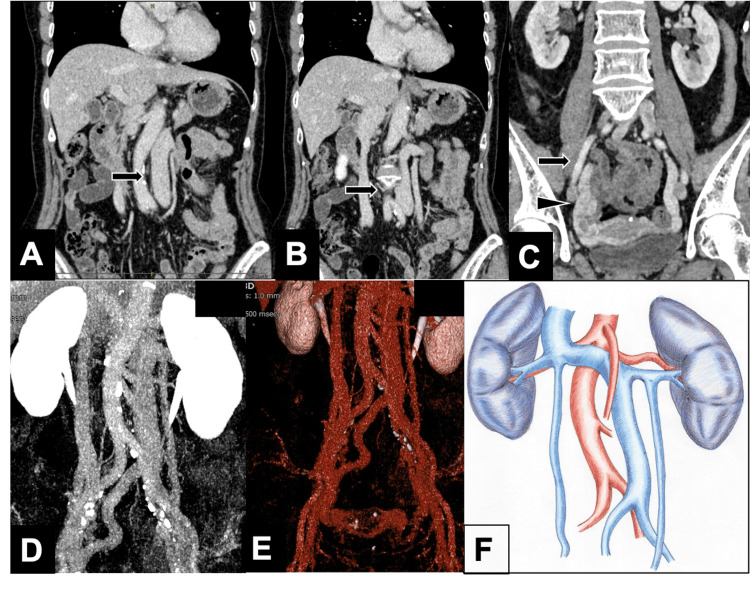
CT findings of a left-sided inferior vena cava A: CT revealed that the inferior vena cava (IVC) was located on the left side of the aorta (arrow), leading to a diagnosis of left-sided IVC. B: The right ovarian vein was mildly dilated and drained into the left-sided IVC (arrow). C: CT imaging showing no clear communication between the right iliac vein (arrow) and the right ovarian vein (arrowhead), despite their anatomical proximity. D, E: Three-dimensional reconstructed CT image showing the IVC located on the left side of the aorta, confirming the diagnosis of left-sided IVC. F: Schematic diagram illustrating the left-sided IVC. Image Credit: This original illustration was hand-drawn by author Naoki Rikiyama.

**Figure 3:**
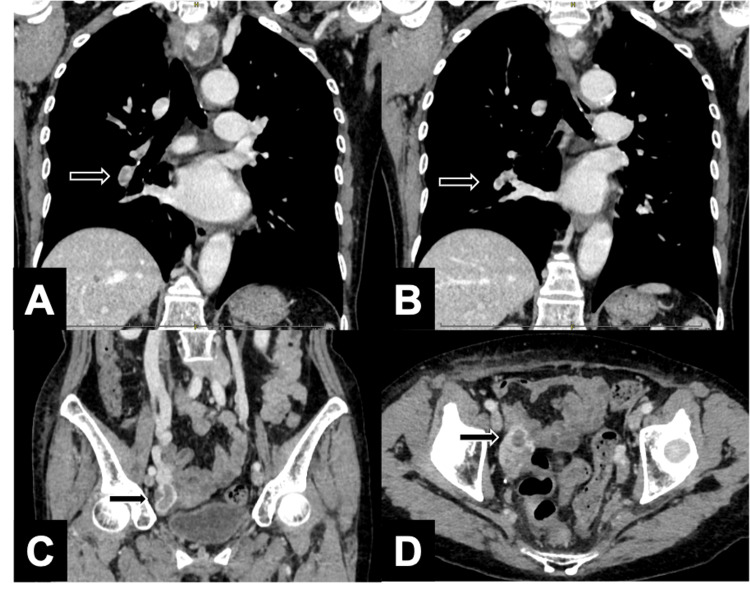
Postoperative contrast-enhanced CT findings A, B: Contrast-enhanced CT demonstrated filling defects in the pulmonary arteries (arrows), consistent with pulmonary thromboembolism (PTE). C, D: Postoperative CT revealed a thrombus in the right ovarian vein (arrows), consistent with ovarian vein thrombosis (OVT).

A 79-year-old woman, who was under surveillance for gallbladder adenomyomatosis (ADM), showed progressive thickening of the gallbladder wall on a follow-up computed tomography (CT) (Figure 1A). Endoscopic ultrasonography (EUS) raised suspicion of gallbladder carcinoma (Figure 1B). Endoscopic retrograde cholangiopancreatography (ERCP) revealed a pancreaticobiliary maljunction without significant dilation of the common bile duct (Figure 1C). The amylase level of the bile collected during the ERCP was 200,111 IU/L. Although bile cytology did not show definitive evidence of malignancy, gallbladder carcinoma could not be ruled out, and the patient was thus referred to our department for surgical evaluation. Positron emission tomography with fluorodeoxyglucose (FDG-PET) revealed mild tumor uptake without evidence of distant metastasis (Figure 1D). Preoperative CT demonstrated the presence of a left-sided IVC (Figures 2A, 2D-2F) and slightly dilated right ovarian vein (Figures 2B, 2D, 2E). There was no clear communication between the right iliac vein and the right ovarian vein (Figure 2C). Based on the ninth edition of the Union for International Cancer Control (UICC) TNM classification [10], the preoperative diagnosis was T2bN0M0, Stage IIa. Laparoscopic gallbladder bed resection and lymphadenectomy were performed following lymph node sampling at station #16b1. Intraoperative rapid pathological evaluation of peritoneal cytology and station #16b1 lymph node sampling were negative. Therefore, laparoscopic gallbladder bed resection with lymphadenectomy was completed as planned. During sampling of lymph node #16b1, the left-sided IVC was exposed; however, the right ovarian vein was not identified, as extensive dissection was deemed unnecessary. The surgery was conducted in the reverse Trendelenburg position, lasting 268 min with an estimated blood loss of 73 mL. The initial postoperative course of the patient was uneventful. However, a routine surveillance CT on postoperative day five unexpectedly revealed PTE (Figures 3A, 3B) and right OVT (Figure 3C, 3D). Based on these findings, the patient was diagnosed with PTE secondary to right OVT in the context of left-sided IVC. Anticoagulant therapy was initiated with heparin and later transitioned to rivaroxaban. The patient was discharged on postoperative day 13 without further complications. A CT scan before discharge showed a reduction in PTE; however, OVT remained largely unchanged. Final pathology revealed a hyperplastic polyp with ADM, but no evidence of carcinoma.

## Discussion

OVT is a rare but clinically significant condition that involves thrombus formation in the ovarian veins and can potentially lead to PTE [9,11]. While OVT is typically associated with the postpartum period, particularly after cesarean delivery, and has an incidence of 0.05-0.18% [5], it is not widely recognized as a complication of gastrointestinal surgery [6]. A recent cohort study emphasizes that symptomatic postpartum OVT presents with a distinct profile of abdominal pain and fever [7]. Furthermore, OVT can be easily missed when it mimics other postoperative conditions, necessitating a high index of clinical suspicion for early diagnosis [7]. The right ovarian vein is more frequently affected (80-90%) due to its anatomical and physiological features, including its direct drainage into the IVC and the presence of an incomplete valve mechanism [12,13]. Other risk factors for OVT include the postpartum state, pelvic infections, malignancies, coagulation disorders, pelvic surgeries, trauma, and venous stasis [5]. Contrast-enhanced CT is the primary imaging modality for diagnosis and typically reveals ovarian vein dilation and thrombus formation [13,14]. Treatment primarily involves anticoagulation therapy, typically initiated with intravenous heparin followed by oral anticoagulants, including direct oral anticoagulants (DOACs), for three to six months [7]. Broad-spectrum antibiotics are recommended for cases complicated by infection. Surgical intervention may be required if anticoagulation therapy is ineffective. Although the prognosis is generally favorable with appropriate treatment, the risk of PTE underscores the need for early diagnosis and intervention. Long-term anticoagulation may be necessary in patients with underlying coagulopathies [14-16].

Left-sided IVC is a rare anatomical variant, found in approximately 0.2-0.5% of the population. It results from persistence of the left posterior cardinal vein during embryogenesis and may be accompanied by a duplicated IVC [2]. Although often asymptomatic and discovered incidentally during imaging or surgery, left-sided IVC has important clinical implications, especially during abdominal and pelvic surgery or vascular interventions. This anomaly increases the risk of vascular injury, particularly to the left renal vein, and may predispose patients to DVT and PTE due to altered venous hemodynamics. In the context of OVT, such anatomical variations can further complicate venous return and promote stasis, a key component of Virchow’s triad. In our case, the left-sided IVC likely resulted in an unusually long and horizontal course for the right ovarian vein to reach the midline, potentially exacerbating venous congestion [8]. Additionally, atypical venous anatomy can complicate the diagnosis of venous thrombosis, highlighting the importance of preoperative imaging. In cases of DVT associated with left-sided IVC, thrombi often occur in the iliac and femoral veins, distal to where the IVC crosses the aorta [17]. Complications of left-sided IVC include an increased risk of PTE and chronic venous insufficiency, which may present as limb edema or varicosities. In rare cases, surgical resection or venous stenting may be required to restore adequate venous return [5].

Although rare, gallbladder tumors present diagnostic and therapeutic challenges due to their frequently asymptomatic presentation in early stages [18]. In the present case, laparoscopic resection allowed for accurate tumor margin assessment and successful removal, while preserving surrounding tissue, minimizing postoperative pain and infection risk, and promoting faster recovery. Although the final pathology was benign, early gallbladder carcinoma is notoriously difficult to diagnose preoperatively. Laparoscopic gallbladder bed resection is a feasible and oncologically appropriate approach in such cases, allowing for tumor excision and lymph node dissection with minimal invasiveness. The role of laparoscopic resection in early-stage gallbladder cancer is expected to grow. Additionally, robot-assisted surgery, with enhanced precision and a minimally invasive approach, may further improve outcomes and recovery.

A comprehensive PubMed search revealed no previously reported cases of OVT associated with left-sided IVC. Moreover, no documented cases of DVT in left-sided IVC occurring during laparoscopic cholecystectomy were found, making this the first such case reported in the literature. The incidence of DVT after laparoscopic cholecystectomy is less than 1%, and routine perioperative prophylactic anticoagulation is generally not recommended [19].

In this case, in addition to the left-sided IVC potentially causing venous stasis in the right ovarian vein, where the right ovarian vein followed an unusually long and horizontal course to reach the midline IVC, surgical manipulation near the IVC during station #16b1 lymph node sampling may have contributed to OVT by inducing localized endothelial trauma. During #16b1 lymph node sampling, careful hemostasis is necessary due to the abundance of lymphatics and small vessels. While an ultrasonic coagulating and cutting device is effective, it carries a risk of thermal injury to nearby vascular structures. We emphasize that meticulous surgical technique, with careful attention to minimizing both thermal and mechanical damage to surrounding vessels, is more critical than the choice of a specific device. Postoperative inflammation likely further enhanced the prothrombotic state. Although a retrospective review of the surgical video did not explicitly identify the right ovarian vein, the dissection was performed in close proximity to its expected anatomical location. Therefore, the possibility of unintended thermal injury or surgical manipulation to the venous wall cannot be entirely ruled out as a contributing factor for endothelial damage. The extended surgery duration and use of reverse Trendelenburg positioning may have exacerbated venous congestion in the lower body, including the ovarian veins. Although OVT is well-documented in gynecological surgery, its occurrence in gastrointestinal procedures, especially resulting in PTE, is exceedingly rare. Thus, this case is both exceptional and clinically meaningful. 

This finding emphasizes the importance of recognizing left-sided IVC as a potential risk factor for venous thromboembolism in abdominal surgery. Preoperative imaging is essential for identifying vascular anomalies and guiding intraoperative planning. Moreover, heightened vigilance for OVT is warranted in patients with left-sided IVC, even after minimally invasive surgery. Although perioperative thromboprophylaxis was not administered in this case, postoperative prophylactic anticoagulation should be considered in abdominal surgeries involving left-sided IVC due to the increased thrombotic risk. Furthermore, prophylactic ligation or division of the right ovarian vein could be a potential option, particularly in high-risk cases, such as those involving extensive retroperitoneal dissection or intraoperative findings of significant venous congestion, provided the patient accepts the potential risks and benefits.

## Conclusions

We report a rare case of gallbladder tumor in a patient with left-sided IVC complicated by OVT, resulting in PTE. This unique clinical presentation highlights the importance of thorough preoperative evaluation, meticulous intraoperative techniques, and vigilant postoperative monitoring. To our knowledge, this is the first reported case of PTE secondary to OVT in the context of left-sided IVC following laparoscopic gallbladder bed resection, underscoring its rarity and potential clinical significance. Identifying anatomical variants, such as left-sided IVC, and evaluating thrombotic risk may help prevent severe complications, even in minimally invasive gastrointestinal surgery.
